# Chorea : Rheumatism of the Brain

**Published:** 1901-10-26

**Authors:** 


### CHOREA: RHEUMATISM OF THE BRAIN.

Not merely does Sir Dyce Duckworth[1] agree with those writers who maintain the frequency of rheumatism as an antecedent or concomitant of chorea, but he goes a step further and holds that chorea is in fact rheumatism of the brain, and in regard to the many other diseases and circumstances which have from time to time been noted as antecedents and apparent causes of chorea, he holds them as only influences by which the resisting power, local or general as the case may be, has been lowered, thus allowing the pre-existing rheumatic tendency to come into play. With respect to all these conditions he says: "I would venture to suggest that the subject of chorea will be found to be of the rheumatic habit, and that all these alleged provocatives are not the specific determinants of the disorder." So far as nervous causes are concerned, it must be remembered that the effects of fright in certain people, especially children, are profound and very grave. One effect is probably to lower the trophic rhythm of the nervous system and to lay it open to immediate invasion by any malign or infective influence that may be at hand, as a *locus minoris resistentiæ*. By fright, or strain, or emotion, then, the brain is rendered specially vulnerable and sensitive to the effects of the rheumatic infection, which alights on the brain-cells and neurons, instead of on the throat, the joints, or the different cardiac textures, as in ordinary cases of acute rheumatism. Chorea, then, is rheumatism of the brain.

[1] Clin. Jour., Oct. 2.

